# Immune reconstitution inflammatory syndrome (IRIS): review of common infectious manifestations and treatment options

**DOI:** 10.1186/1742-6405-4-9

**Published:** 2007-05-08

**Authors:** David M Murdoch, Willem DF Venter, Annelies Van Rie, Charles Feldman

**Affiliations:** 1 Division of Pulmonary and Critical Care Medicine, Duke University Medical Center, Durham North Carolina, USA; 2 Reproductive Health & HIV Research Unit, University of the Witwatersrand, Johannesburg, South Africa; 3 Department of Epidemiology, The University of North Carolina at Chapel Hill, Chapel Hill, NC, USA; 4 Division of Pulmonology, Department of Medicine, Johannesburg Hospital and University of the Witwatersrand, Johannesburg, South Africa; 5 CB#7435, 2104-H McGavran-Greenberg Hall, University of North Carolina, School of Public Health, Chapel Hill, NC 27599-7435, USA

## Abstract

The immune reconstitution inflammatory syndrome (IRIS) in HIV-infected patients initiating antiretroviral therapy (ART) results from restored immunity to specific infectious or non-infectious antigens. A paradoxical clinical worsening of a known condition or the appearance of a new condition after initiating therapy characterizes the syndrome. Potential mechanisms for the syndrome include a partial recovery of the immune system or exuberant host immunological responses to antigenic stimuli. The overall incidence of IRIS is unknown, but is dependent on the population studied and its underlying opportunistic infectious burden. The infectious pathogens most frequently implicated in the syndrome are mycobacteria, varicella zoster, herpesviruses, and cytomegalovirus (CMV). No single treatment option exists and depends on the underlying infectious agent and its clinical presentation. Prospective cohort studies addressing the optimal screening and treatment of opportunistic infections in patients eligible for ART are currently being conducted. These studies will provide evidence for the development of treatment guidelines in order to reduce the burden of IRIS. We review the available literature on the pathogenesis and epidemiology of IRIS, and present treatment options for the more common infectious manifestations of this diverse syndrome and for manifestations associated with a high morbidity.

## Introduction

Since its introduction, ART has led to significant declines in AIDS-associated morbidity and mortality [1]. These benefits are, in part, a result of partial recovery of the immune system, manifested by increases in CD4^+ ^T-lymphocyte counts and decreases in plasma HIV-1 viral loads [2]. After initiation of ART, opportunistic infections (OI) and other HIV-related events still occur secondary to a delayed recovery of adequate immunity [3].

Some patients initiating ART experience unique symptoms during immune system recovery. In these patients, clinical deterioration occurs despite increased CD4^+ ^T-lymphocyte counts and decreased plasma HIV-1 viral loads [4]. This clinical deterioration is a result of an inflammatory response or "dysregulation" of the immune system to both intact subclinical pathogens and residual antigens [5-9]. Resulting clinical manifestations of this syndrome are diverse and depend on the infectious or noninfectious agent involved. These manifestations include mycobacterial-induced lymphadenitis [5], paradoxical tuberculosis reactions [6,7,10,11], worsening of progressive multifocal leukoencephalopathy (PML) [12], recurrence of cryptococcosis and *Pneumocystis jirovecii *pneumonia (PCP) [8,13-16], *Cytomegalovirus *(CMV) retinitis [17], shingles [18], and viral hepatitis [19], as well as noninfectious phenomena [20].

Because clinical deterioration occurs during immune recovery, this phenomenon has been described as immune restoration disease (IRD), immune reconstitution syndrome (IRS), and paradoxical reactions. Given the role of the host inflammatory response in this syndrome, the term immune reconstitution inflammatory syndrome (IRIS) has been proposed [21] and has become the most widely used and accepted term to describe the clinical entity. Possible infectious and noninfectious etiologies of IRIS are summarized in Table 1.

**Table 1:** Infectious and noninfectious causes of IRIS in HIV-infected patients

**Infectious Etiologies**	**Noninfectious etiologies**
Mycobacteria	Rheumatologic/Autoimmune
*Mycobacterium tuberculosis *[4, 6, 7, 10, 11, 26, 30-32, 41, 43, 45]	Rheumatoid arthritis [29] Systemic lupus erythematosus (SLE) [91]
	Graves disease [92], Autoimmune thyroid disease [93]
*Mycobacterium avium *complex [4, 5, 23, 31, 94-96]	Sarcoidosis & granulomatous reactions [20, 97]
Other mycobacteria [4, 56, 57, 98, 99]	Tattoo ink [100]
*Cytomegalovirus *[4, 33, 61, 63]	AIDS-related lymphoma [101]
Herpes viruses	Guillain-Barre' syndrome (GBS) [102]
Herpes zoster virus [4, 32, 33, 71, 103, 104]	Interstitial lymphoid pneumonitis [105]
Herpes simplex virus [4, 32, 33]	
Herpes virus-associated Kaposi's sarcoma [4, 32, 106]	
*Cryptococcus neoformans *[13, 16, 22, 28, 31, 83, 84, 86, 88]	
*Pneumocystis jirovecii *pneumonia (PCP) [8, 14, 32]	
*Histoplasmosis capsulatum *[107]	
Toxoplasmosis [33]	
Hepatitis B virus [32, 33]	
Hepatitis C virus [4, 32, 33, 108]	
Progressive multifocal leukoencephalitis [12, 33, 109]	
Parvovirus B19 [110]	
*Strongyloides stercoralis *infection [111] & other parasitic infections [112]	
Molluscum contagiosum & genital warts [32]	
Sinusitis [113]	
Folliculitis [114, 115]	

To date, no prospective therapeutic trials concerning the management of IRIS have been conducted. All evidence regarding the management of IRIS in the literature relates to case reports and small case series reporting on management practice. This does not provide reliable evidence regarding either the safety or efficacy of these approaches, but merely guidance regarding the practice of others in managing this difficult condition. In severe cases where the discontinuation of ART is a possibility, the potential disadvantages of therapy cessation, such as the development of viral resistance or AIDS progression, should be considered.

### Pathogenesis of IRIS

Despite numerous descriptions of the manifestations of IRIS, its pathogenesis remains largely speculative. Current theories concerning the pathogenesis of the syndrome involve a combination of underlying antigenic burden, the degree of immune restoration following HAART, and host genetic susceptibility. These pathogenic mechanisms may interact and likely depend on the underlying burden of infectious or noninfectious agent.

Whether elicited by an infectious or noninfectious agent, the presence of an antigenic stimulus for development of the syndrome appears necessary. This antigenic stimulus can be intact, "clinically silent" organisms or dead or dying organisms and their residual antigens. IRIS that occurs as a result of "unmasking" of clinically silent infection is characterized by atypical exuberant inflammation and/or an accelerated clinical presentation suggesting a restoration of antigen-specific immunity. These characteristics differentiate IRIS from incident opportunistic infections that occur on ART as a result of delayed adequate immunity.

Examples of IRIS in response to intact organisms include, but are not limited to, the unmasking of latent cryptococcal infection [22] and infection with *Mycobacterium avium *complex (MAC) [4,5,23,24]. The most frequently reported IRIS symptoms in response to previously treated or partially treated infections include reports of clinical worsening and recurrence of clinical manifestations of *Mycobacterium tuberculosis *(TB) and cryptococcal meningitis following initiation of ART [6,7,10,13,16,25-28]. In noninfectious causes of IRIS, autoimmunity to innate antigens plays a likely role in the syndrome. Examples include exacerbation of rheumatoid arthritis and other autoimmune diseases [29]. Given the role of this antigenic stimulus, the frequency and manifestations of IRIS in a given population may be determined by the prevalence of opportunistic and non-opportunistic infections to initiation of ART.

The mechanism receiving the most attention involves the theory that the syndrome is precipitated by the degree of immune restoration following ART. In assessing this theory, investigators have examined the association between CD4 cell counts and viral loads and the risk of IRIS. Some studies suggest differences in the baseline CD4 profiles or quantitative viral load at ART initiation or their rate of change during HAART between IRIS and non-IRIS patients [4,30-34], while other studies demonstrate only trends or no significant difference between IRIS and non-IRIS patients [7,35]. These immunological differences between groups have been difficult to verify due to small numbers of IRIS cases and lack of control groups. An alternative immunological mechanism may involve qualitative changes in lymphocyte function or lymphocyte phenotypic expression. For instance, following ART an increase in memory CD4 cell types is observed [36] possibly as a result of redistribution from peripheral lymphoid tissue [37]. This CD4 phenotype is primed to recognize previous antigenic stimuli, and thus may be responsible for manifestations of IRIS seen soon after ART initiation. After this redistribution, naïve T cells increase and are thought to be responsible for the later quantitative increase in CD4 cell counts [38]. These data suggest IRIS may be due to a combination of both quantitative restoration of immunity as well as qualitative function and phenotypic expression observed soon after the initiation of ART.

The third purported pathogenic mechanism for IRIS involves host genetic susceptibility to an exuberant immune response to the infectious or noninfectious antigenic stimulus upon immune restoration. Although evidence is limited, carriage of specific HLA alleles suggest associations with the development of IRIS and specific pathogens [39]. Increased levels of interleukin-6 (IL-6) in IRIS patients may explain the exuberant Th1 response to mycobacterial antigens in subjects with clinical IRIS [9,40]. Such genetic predispositions may partially explain why manifestations of IRIS differ in patients with similar antigenic burden and immunological responses to ART.

### Epidemiology of IRIS

Despite numerous descriptions of the infectious and noninfectious causes of IRIS, the overall incidence of the syndrome itself remains largely unknown. Studies to date are often retrospective and focus on specific manifestations of IRIS, such as tuberculosis-associated IRIS (TB-IRIS). In a large retrospective analysis examining all forms of IRIS, 33/132 (25%) of patients exhibited one or more disease episodes after initiation of ART [4]. Other cohort analyses examining all manifestations of IRIS estimate that 17–23% of patients initiating ART will develop the syndrome [32-34]. Another large retrospective study reported 32% of patients with *M. tuberculosis, M. avium *complex, or *Cryptococcus neoformans *coinfection developed IRIS after initiating ART.

Risk factors identified for the development of IRIS in one cohort included male sex, a shorter interval between initiating treatment for OI and starting ART, a rapid fall in HIV-1 RNA after ART, and being ART-naïve at the time of OI diagnosis [31]. Other significant predictors have also included younger age, a lower baseline CD4 cell percentage, a lower CD4 cell count at ART initiation, and a lower CD4 to CD8 cell ratio at baseline [4,32]. It should be noted cohorts differ substantially in study populations and the type of IRIS (i.e. TB-IRIS only) examined, making conclusions regarding risk factors for IRIS difficult. Clinical factors associated with the development of IRIS are presented in Table 2.

**Table 2:** Clinical factors associated with the development of IRIS^†^

Risk factor	Reference
Male sex	[31]
Younger age	[32]
Lower CD4 cell count at ART initiation	[4]
Higher HIV RNA at ART initiation	[4]
Lower CD4 cell percentage at ART initiation	[32]
Lower CD4:CD8 ratio at ART initiation	[32]
More rapid initial fall in HIV RNA on ART	[31]
Antiretroviral naïve at time of OI diagnosis	[31]
Shorter interval between OI therapy initiation and ART initiation	[31]

^†^Derived from cohorts where IRIS due to multiple pathogens were reported (i.e. cohorts which examined only TB-IRIS were excluded)

Case reports describing different clinical manifestations of IRIS continue to appear, expanding the clinical spectrum of the syndrome. Because the definition of IRIS is one of clinical suspicion and disease-specific criteria have yet to be developed, determining the true incidence will be difficult. Taken together, these studies suggest IRIS may affect a substantial proportion of HIV patients initiating ART. Future epidemiologic and genetic studies conducted within diverse cohorts will be important in determining the importance of host susceptibility and underlying opportunistic infections on the risk of developing IRIS.

### Disease-specific manifestations of IRIS

In order to aid clinicians in the management of IRIS, we review the epidemiology, clinical features, and treatment options for the common infectious manifestations of IRIS. Additionally, manifestations associated with significant morbidity and mortality, such as CMV-associated immune recovery vitritis (IRV) or immune recovery uveitis (IRU), are also reviewed. Treatment options and their evidence are presented. Until disease specific guidelines are developed for IRIS, therapy should be based on existing evidence and individualized according to the severity of presentation.

### Mycobacterium tuberculosis IRIS

#### Epidemiology

*Mycobacterium tuberculosis *(TB) is among the most frequently reported pathogen associated with IRIS. Narita *et al *performed the first prospective study to evaluate the incidence of paradoxical responses in patients on TB therapy and subsequently initiated on ART. Of 33 HIV/TB coinfected patients undergoing dual therapy, 12 (36%) developed paradoxical symptoms [7]. The frequency of symptoms in this group were greater than those observed in HIV-infected controls receiving TB therapy alone, supporting the role of an exaggerated immune system response in the pathogenesis of the syndrome. Retrospective studies corroborate the finding that a significant proportion of HIV/TB coinfected patients undergoing HAART have symptoms consistent with IRIS, with estimates ranging from 7–45% [10,26,30,35,41-43].

The association between a shorter delay between TB treatment initiation and ART initiation is an area of debate. While some investigators have found no difference in time from TB therapy to initiation of ART between IRIS and non-IRIS subjects [30], others have reported a significant differences between groups [31,35]. In general, IRIS occurred in subjects initiated on ART within two months of TB therapy initiation [35]. Based on these and other data, a decision analysis on ART initiation timing in TB patients found the highest rates of IRIS occurred in patients initiated on ART within two months of TB therapy initiation [44]. However, withholding or deferring ART until two to six months of TB therapy was associated with higher mortality in scenarios where IRIS-related mortality was less than 4.6%. Future reports from large, prospective observational cohorts may aid in resolving this difficult issue.

Although consisting primarily of case reports [45,46], TB-IRIS affecting the central nervous system (CNS) poses a unique problem. As the availability of ART increases in endemic countries, the incidence of CNS TB-IRIS may increase. Thus, clinicians should be vigilant in its diagnosis.

#### Clinical features

The commonest clinical manifestations of TB-IRIS are fever, lymphadenopathy and worsening respiratory symptoms [47]. Pulmonary disorders, such as new pulmonary infiltrates, mediastinal lymphadenopathy, and pleural effusions are also common [7]. Extrapulmonary presentations are also possible, including disseminated tuberculosis with associated acute renal failure [6], systemic inflammatory responses (SIRS) [48], and intracranial tuberculomas [45]. Pulmonary TB-IRIS can be diagnosed by transient worsening of chest radiographs, especially if old radiographs are available for comparison. Other symptoms are nonspecific, and include persistent fever, weight loss, and worsening respiratory symptoms. Abdominal TB-IRIS can present with nonspecific abdominal pain and obstructive jaundice.

In most studies, TB-IRIS occurs within two months of ART initiation [6,7,10,11,25,35,45,48]. Among 43 cases of MTB-associated IRIS, the median onset of IRIS was 12–15 days (range 2–114 days), with only four of these cases occurring more than four weeks after the initiation of antiretroviral therapy [7,10,25,26,30]. These studies suggest the onset of mycobacterial-associated IRIS is relatively soon after initiation of ART, and clinicians should maintain a high level of vigilance during this period.

Paradoxical CNS TB reactions are well described in HIV-negative patients, and include expanding intracranial tuberculomas, tuberculous meningitis, and spinal cord lesions [49-51]. TB-associated CNS IRIS has also been reported in HIV-positive patients [45,46,52]. Compared to non-CNS TB-IRIS, symptoms tend to occur later, usually 5–10 months after ART initiation [45,50,52]. Crump et al [45] described an HIV-seropositive patient in who developed cervical lymphadenopathy after five weeks of ART. Five months later, CNS symptoms associated with an expanding intracranial tuberculoma appeared after initiation of antituberculous therapy. The significant morbidity in this case illustrates the importance of maintaining a high clinical suspicion for the disease, particularly in endemic areas.

#### Treatment

Treatment for mycobacterial-associated IRIS depends on the presentation and disease severity. Most patients present with non-life threatening presentations which respond to the institution of appropriate antituberculous therapy. However a range of life threatening presentations, such as acute renal failure [6] and acute respiratory distress syndrome (ARDS) [11], are described and have significant morbidity and mortality. Morbidity and mortality might also be greater in resource-limited settings where limited management options exist. Since the pathogenesis of the syndrome is an inflammatory one, systemic corticosteroids or nonsteroidal anti-inflammatory drugs (NSAIDS) may alleviate symptoms. In studies where therapy for IRIS was mentioned, the use of corticosteroids was variable [7,24,25,31,41,43] and anecdotally effective. Therapies ranged from intravenous methylprednisolone 40 mg every 12 hours to prednisone 20–70 mg/day for 5–12 weeks. These practices reflect the lack of evidence from controlled trials for the use of anti-inflammatory agents in IRIS. A randomized, placebo controlled trial examining doses of prednisone 1.5 mg/kg/day for two weeks followed by 0.75 mg/kg/day for two weeks in mild to moderate TB-IRIS is currently underway in South Africa. Until data become available, it is reasonable to administer corticosteroids for severe cases of IRIS such as tracheal compression due to lymphadenopathy, refractory or debilitating lymphadenitis, or severe respiratory symptoms, such as stridor and ARDS. Interruption of ART is rarely necessary but could be considered in life-threatening situations.

In HIV-negative patients, adjuvant corticosteroid use in tuberculous meningitis provides evidence of improved survival and decreased neurologic sequelae over standard therapy alone [53,54]. Once other infectious etiologies, have been excluded, standard antituberculous therapy should be initiated or continued as the clinical situation dictates, and a course of corticosteroid therapy should be considered for CNS TB-IRIS. Continuation of ART is desirable, although its discontinuation may be necessary in unresponsive cases or in those presenting with advanced neurological symptoms.

### Atypical mycobacterial IRIS

#### Epidemiology

In addition to TB, atypical mycobacteria are also frequently reported as causative pathogens in IRIS. Early observations involving atypical presentations of *Mycobacterium avium-intracellulare *(MAC) were first noted with zidovudine therapy [55]. Reports of atypical presentations of both *Mycobacterium tuberculosis *(MTB) and MAC increased in frequency with the introduction of protease inhibitors and ART. In larger cohorts, MAC remains the most frequently reported atypical mycobacterium [4,5,24]. Other atypical mycobacteria rarely associated with IRIS are referenced in Table 1.

#### Clinical features

In general, MAC-associated IRIS typically presents with lymphadenitis, with or without abscess formation and suppuration [5]. Other less common presentations include respiratory failure secondary to acute respiratory distress syndrome (ARDS) [56], leprosy [57], pyomyositis with cutaneous abscesses [23], intra-abdominal disease [58], and involvement of joints, skin, soft tissues, and spine [58,59].

Several studies have characterized the time of onset of *Mycobacterium*-associated IRIS. In one study of MAC lymphadenitis, the onset of a febrile illness was the first sign of IRIS and occurred between 6 and 20 days after initiation of antiretroviral therapy [5]. In another study, the median time interval from the start of antiretroviral therapy to the development of mycobacterial lymphadenitis was 17 days (range 7–85 days) [24].

#### Treatment

As with TB-IRIS, evidence for treatment of IRIS due to atypical mycobacteria are scarce. Occasionally, surgical excision of profoundly enlarged nodes or debridement of necrotic areas is anecdotally reported [23,59]. However, healing is often poor leaving large, persistent sinuses. Needle aspiration is another option for enlarged, fluctuant and symptomatic nodes. Otherwise, treatment is similar to TB-IRIS (see Mycobacterium tuberculosis IRIS – Treatment).

### Cytomegalovirus infection

#### Epidemiology

In the pre-ART era, CMV retinitis, a vision-threatening disease, carried a high annual incidence and was one of the most significant AIDS-associated morbidities [60]. After the introduction of HAART, Jacobson et al described five patients diagnosed with CMV retinitis 4–7 weeks after ART initiation. They speculated that an HAART-induced inflammatory response may be responsible for unmasking a subclinical infection [17]. In addition to classical CMV retinitis, ART led to new clinical manifestations of the infection, termed immune recovery vitritis (IRV) or immune recovery uveitis (IRU), in patients previously diagnosed with inactive AIDS-related CMV retinitis [61]. Distinct from the minimal intraocular inflammation of classic CMV retinitis, these manifestations exhibit significant posterior segment ocular inflammation thought to be due to the presence of residual CMV antigens or proteins which serve as the antigenic stimulus for the syndrome [62]. Clinical manifestations include vision impairment and floaters.

In a retrospective cohort, CMV-related IRIS was common (6/33 of IRIS cases, or 18%) [4]. In prospective cohorts, symptomatic vitritis occurred in 63% (incidence rate 83 per 100 p-yr) of ART responders who carried a previous diagnosis of CMV retinitis but had inactive disease at the onset of antiretroviral therapy. The median time from ART initiation to IRV was (43 weeks)[63]. Another large prospective surveillance study [64] identified 374 patients with a history of CMV retinitis involving 539 eyes. Thirty-one of 176 ART responders (17.6%) were diagnosed with IRU. Male gender, use of ART, higher CD4 cell counts, and involvement of the posterior retinal pole as factors associated with a reduced risk of developing IRU, whereas prior use of intravitreous injections of cidofovir, large retinal lesions, and adequate immune recovery on ART were associated with increased risk.

#### Clinical features & treatment

The diagnosis of ocular manifestations of IRIS requires a high level of suspicion. In addition to signs of retinitis, inflammatory symptoms include vitritis, papillitis, and macular edema, resulting in symptoms of loss of visual acuity and floaters in affected eyes. Treatment of IRIS associated CMV retinitis and IRV may involve anti-CMV therapy with gancyclovir or valgancyclovir[17,65]. However, the occurrence of IRU in patients receiving anti-CMV therapy draws its use into question [64,66,67]. The use of systemic corticosteroids has been successful, and IRV may require periocular corticosteroid injections [61,68-70]. Due to its significant morbidity and varying temporal presentations, clinicians should maintain a high level of vigilance for ocular manifestations of CMV-associated IRIS.

### Varicella zoster virus infection

#### Epidemiology

With the introduction of protease inhibitors, increasing rates of herpes zoster were noted in HIV-infected patients. Two studies comparing ART and non-ART patients reported increased incident cases of zoster and rates estimated at 6.2–9.0 cases per 100 person-years, three to five times higher than rates observed in the pre-HAART era [18,71]. While another study [72] reported no difference in overall incidence between HAART eras (3.2 cases per 100 person-years), the use of HAART was associated with increased odds of developing an incident zoster outbreak (OR = 2.19, 95% confidence interval: 1.49 to 3.20). These studies suggest that ART may play a role in increasing the risk of zoster, which is reflected in large observational IRIS cohorts, where dermatomal varicella zoster comprises 9–40% of IRIS cases [4,32,33]. Mean onset of disease from ART initiation was 5 weeks (range 1–17 weeks) [71], and no cases occurred before 4 weeks of therapy [18]. Both studies identified significant increases in CD8 T cells as a risk factor for developing dermatomal zoster.

#### Clinical features & treatment

Although complications such as encephalitis, myelitis, cranial and peripheral nerve palsies, and acute retinal necrosis can occur in immunocompromised HIV patients, the vast majority of patients exhibit typical or atypical dermatomal involvement without dissemination or systemic symptoms [18,71,73].

A randomized, controlled trial demonstrated oral acyclovir to be effective for dermatomal zoster in HIV-infected patients, facilitating healing and shortening the time of zoster-associated pain [74]. Its use in cases of varicella zoster IRIS appears to be of clinical benefit [18]. The benefit of corticosteroids in combination with acyclovir in acute varicella zoster has been demonstrated in two large randomized, controlled trials. The combination of corticosteroids and acyclovir decreased healing times, improved acute pain, and quality of life, but did not affect the incidence or duration of postherpetic neuralgia [75,76]. The incidence of postherpetic neuralgia in immunocompetent individuals does not differ significantly from HIV-infected patients, but increases with increasing patient age [77]. Successful symptomatic management involving opioids, tricyclic antidepressants, gabapentin, and topical lidocaine patches individually or in combination has been shown to be beneficial [78-82] and should be attempted in HIV patients with postherpetic neuralgia as a complication of herpes zoster IRIS.

### Cryptococcus neoformans infection

#### Epidemiology

Accurate incidence of *C. neoformans*-associated IRIS is unknown. It is infrequently reported in overall IRIS cohorts, and many cases appear as single case reports. The majority of cryptococcal IRIS cases represent reactivation of previously treated cases [13,16,21,22,83-86], suggesting either an immunological reaction to incompletely treated disease or an inflammatory reaction to residual antigens. Although reports of cryptococcal lymphadenitis and mediastinitis have been reported [87,88], most cryptococcal IRIS cases present as meningitis. Of 41 well documented cases of cryptococcal IRIS meningitis, 33 (80%) result as a reactivation of *C. neoformans *meningitis [13,16,21,22,83-86,89], illustrating the importance of maintaining a high clinical suspicion for patients at risk for cryptococcal IRIS, even in those previously treated.

#### Clinical features

*C. neoformans*-induced IRIS meningitis symptoms range in onset from seven days to ten months after initiation of ART, with 20 (49%) occurring within four weeks of therapy [13,16,21,22,83-86,89]. In one study [85], patients with *C. neoformans*-related IRIS meningitis were compared to typical AIDS-related *C. neoformans *meningitis. Patients with *C. neoformans*-related IRIS meningitis exhibited no difference in clinical presentation. However, *C. neoformans*-related IRIS patients exhibited had higher baseline plasma HIV RNA levels and higher CSF cryptococcal antigen titers, opening pressures, WBC counts, and glucose levels. Additionally, IRIS patients were more likely to have ART initiated within 30 days of previously diagnosed *C. neoformans *meningitis. Most documented cases of *C. neoformans*-induced IRIS meningitis have occurred in patients with CD4 counts <100 cells/mm^3 ^[13,21,83-85,87].

#### Treatment

A recent study [90] evaluated antifungal combination therapies in the treatment of *C. neoformans *meningitis in HIV patients. Although significant log reductions in colony forming units were observed with all combinations, substantial numbers of patients remained culture positive 2 weeks after therapy. It may be important to delay ART until CSF sterility can be achieved with effective antifungal combinations such as amphotericin B and flucytosine. However, the exact timing of ART and whether attaining CSF culture sterility is important in avoiding IRIS is unknown. This is illustrated by cases of reactivation cryptococcal meningitis described in four patients who had received at least four weeks of antifungal therapy prior to ART [13,22,83]. It is reasonable to administer systemic corticosteroids to alleviate unresponsive inflammatory effects, as anecdotal benefits have been observed in these patients [21,84]. Furthermore, serial lumbar punctures may be required to manage persistent CSF pressure elevations in these patients [85,86]. Although continuation of ART has been performed safely [13,84], interruption of antiviral therapy may be necessary in severe or unresponsive cases.

### Other etiologies

Other less common infectious etiologies, as well as noninfectious etiologies, are listed in Table 1. Because these other infectious and non-infectious etiologies are rare, no recommendations exist for their management.

## Conclusion

While exact estimates of incidence are not yet available, IRIS in patients initiating ART has been firmly established as a significant problem in both high and low income countries. Because of wide variation in clinical presentation and the still increasing spectrum of symptoms and etiologies reported, diagnosis remains problematic. Furthermore, no test is currently available to establish an IRIS diagnosis. Standardized disease-specific clinical criteria for common infectious manifestations of the disease should be developed to: 1) identify risk factors for developing the syndrome and 2) optimize the prevention, management of opportunistic infections. Results of trials addressing the optimal timing and duration of treatment of opportunistic infections will assist in developing guidelines for the prevention and management of IRIS. Treatment of IRIS will remain a clinical challenge due to the variety of clinical presentations and the presence of multiple pathogens capable of causing the syndrome. Until a greater understanding of the syndrome is achieved in different regions of the world, clinicians need to remain vigilant when initiating ART and individualize therapy according to known treatment options for the specific infectious agent.

## Competing interests

The author(s) declare that they have no competing interests.

## Authors' contributions

All authors participated in the drafting of the manuscript. All authors read and approved the final manuscript.
